# Cleavage stimulation factor 2 promotes malignant progression of liver hepatocellular carcinoma by activating phosphatidylinositol 3′-kinase/protein kinase B/mammalian target of rapamycin pathway

**DOI:** 10.1080/21655979.2022.2063100

**Published:** 2022-04-12

**Authors:** Meng-Hui Zhang, Jun Liu

**Affiliations:** a Department of Hepatobiliary Surgery and Center of Organ Transplantation, Shandong Provincial Hospital, Cheeloo College of Medicine, Shandong University, Jinan, Shandong, People’s Republic of China; b Department of General Surgery, The Fourth People’s Hospital of Jinan, Jinan, Shandong, People’s Republic of China

**Keywords:** Liver hepatocellular carcinoma, CSTF2, PI3K/AKT/mTOR pathway

## Abstract

Liver hepatocellular carcinoma (LIHC) is the most common type, comprising 75–85% of all liver malignancies. We investigated the roles of cleavage stimulation factor 2 (CSTF2) in LIHC and explored the underlying mechanisms. CSTF2 expression and its association with LIHC patient survival probability were analyzed with The Cancer Genome Atlas. CSTF2 expression in LIHC cells was assessed using western blot and quantitative real-time PCR. Alterations in CSTF2 expression were induced by cell transfection. Cell colony formation, apoptosis, proliferation, invasion, and migration were assessed using colony formation, flow cytometry, 5-ethynyl-2ʹ-deoxyuridine, and transwell assays. Pathway enrichment analysis was performed using gene set enrichment analysis (GSEA). The expression of apoptosis-, metastasis-, and pathway-associated factors was determined via western blot. The pathway rescue assay was further performed using 740Y-P or Wortmannin. CSTF2 upregulation was observed in LIHC tissues and cells. Patients with high CSTF2 expression had a lower probability of overall survival. CSTF2 overexpression enhanced colony formation, proliferation, invasion and migration, while repressing apoptosis in LIHC cells. GSEA revealed that CSTF2 was mainly enriched in the phosphatidylinositol 3′-kinase/protein kinase B/mammalian target of rapamycin (PI3K/AKT/mTOR) pathway. Western blot analysis proved that CSTF2 overexpression activated this pathway. CSTF2 knockdown yielded the opposite effects. 740Y-P, a PI3K activator, reversed the CSTF2 knockdown-triggered effects on cell proliferation, apoptosis, invasion, and migration. Moreover, Wortmannin, a PI3K inhibitor, also reversed the CSTF2 overexpression-induced effects on cell proliferation, apoptosis, invasion, and migration. These results indicated that CSTF2 overexpression might exacerbate the malignant phenotypes of LIHC cells via activation of PI3K/AKT/mTOR pathway.

## Graphical Abstract

**Figure:**
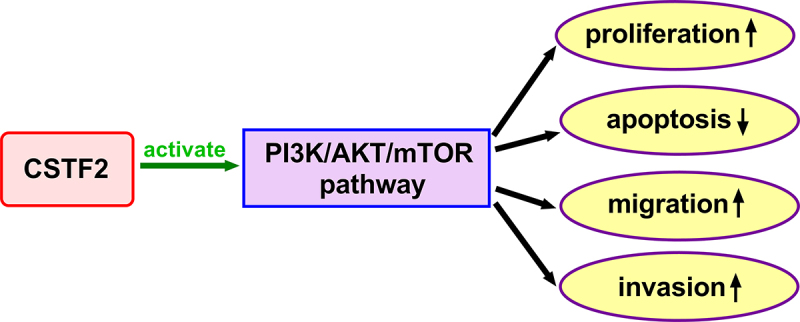

## Introduction

Primary liver cancer is one of the main causes of cancer-associated death in China, and its mortality ranked third among malignant tumors in 2020 [1]. Among all types of primary liver cancer, liver hepatocellular carcinoma (LIHC) is the most common form, which comprises 75–85% of all liver malignancies [2]. Alcohol abuse, hepatitis virus infections, metabolic syndrome, nonalcoholic fatty liver disease, and exposure to aflatoxin B are typical risk factors for LIHC [3,4]. Tumor ablation, surgical resection, liver transplantation, chemotherapy, and sorafenib treatment are the standard therapeutic options [5,6]. However, most patients with LIHC are diagnosed at the middle and late stages, and are not suitable for surgery. Recently, an American study showed that the incidence of LIHC will continue to increase until 2030 [7]. Hence, identifying new early diagnostic biomarkers for LIHC will be of great benefit to patients.

Cleavage stimulation factor (CSTF), which consists of three subunits, CSTF1 (50 kDa), CSTF2 (64 kDa), and CSTF3 (77kDa), is an important modulating component of polyadenylation machinery and mRNA cleavage [8,9]. According to their relative molecular weights, these three subunits are known as CSTF50, CSTF64 and CSTF77, respectively. The CSTF complex exerts pivotal roles in the cleavage reaction by participating in the processing of mRNA 3ʹends [10,11]. It can promote cleavage by binding to the GU/U-rich regions downstream of the cleavage sites [12]. Additionally, CSTF plays a dominant role in the regulation of tumor progression. For instance, CSTF2, as one of the components, was demonstrated to be correlated with poor prognosis of patients with non-small cell lung cancer (NSCLC) [13]. Another study revealed that upregulation of CSTF2 facilitates oncogenic activities in bladder cancer [14]. Zhang *et al*. discovered that CSTF2 was highly expressed in liver cancer tissues [15]. Through retrieving The Cancer Genome Atlas (TCGA)-LIHC database (https://www.genome.gov/Funded-Programs-Projects/Cancer-Genome-Atlas), we found that CSTF2 is highly expressed in LIHC patients, and the patients with high CSTF2 expression had a lower probability of overall survival. All these manifested that CSTF2 may function as an oncogene in LIHC. However, the exact influence of CSTF2 expression on LIHC progression and its potential mechanisms have not yet been reported.

In the current research, we aimed to explore the effect of CSTF2 on cell proliferation, migration, and invasion in LIHC and to investigate the potential underlying molecular mechanisms. First, we analyzed CSTF2 expression and its association with survival probability using The Cancer Genome Atlas. Further, CSTF2 expression in the LIHC cell lines was tested. Based on these results, CSTF2 overexpression and knockdown were carried out, and their effects on proliferation, colony formation, migration, and invasion were detected. Thereafter, pathway enrichment analysis was performed using gene set enrichment analysis (GSEA). The expression of pathway-associated factors was measured, and a pathway rescue assay was conducted. In this study, we confirmed for the first time the effect of CSTF2 on LIHC and suggested that CSTF2 played a critical role in LIHC by modulating the phosphatidylinositol 3′-kinase/protein kinase B/mammalian target of rapamycin (PI3K/AKT/mTOR) pathway. This research provides a theoretical basis for identifying new early diagnostic biomarkers and treatment targets of LIHC.

## Materials and methods

### CSTF2 expression and survival analysis

In this research, CSTF2 expression data and clinical information on LIHC were derived from TCGA database. CSTF2 expression in LIHC and normal tissues was analyzed using gene expression profiling interactive analysis (GEPIA) (http://gepia.cancer-pku.cn/). Kaplan–Meier survival curves were used to explore the influence of CSTF2 expression on the survival of patients with LIHC.

### Cell culture

THLE-3 cells were purchased from the American Type Culture Collection (Rockville, MD, USA). Huh7 cells were obtained from Procell Life Science&Technology Co., Ltd. (Wuhan, China). The Hep-3B, HCCLM3 and MHCC97H cell lines were purchased from Beyotime Biotechnology (Shanghai, China). THLE-3 and Hep-3B cells were cultured in MEM (#E600020), while Huh7, HCCLM3, and MHCC97H cells were cultured in DMEM (#E600004). All media were replenished with 10% fetal bovine serum (FBS, #E600001) and 1 × penicillin–streptomycin solution (#E607011) before use, and were refreshed every 3 days. Cells were cultured under 5% CO_2_ at 37°C. The reagents used in the cell culture were obtained from the BBI Life Sciences Corporation (Shanghai, China).

### Cell transfection and treatment

Huh7 and HCCLM3 cells were cultivated in 6-well plates until they reached 70–80% distribution. Afterward, the original medium was replaced with serum-free DMEM. For the overexpression experiments, Huh7 cells were transfected with the recombinant plasmid pcDNA-CSTF2 and its negative control pcDNA-NC. For the knockdown assay, HCCLM3 cells were transfected with small interfering (si)-CSTF2#1, si-CSTF#2, and negative control si-NC. The recombinant plasmids and siRNA sequences used were were obtained from Gene Pharma (Shanghai, China). Cell transfection was performed using Lipofectamine^TM^ 2000 reagent (#11,668,019, Invitrogen, CA, USA). The culture medium was replaced with complete DMEM at 6 h after transfection. Cell collection was conducted 48 h post transfection.

740Y-P and Wortmannin were obtained from MedChemExpress (#1,236,188-16-1) and Abcam (#ab120148). Transfected HCCLM3 or Huh7 cells were treated with 740Y-P (10 μM) or Wortmannin (10 μM) for 24 h before further analysis.

### Quantitative real-time PCR (qRT-PCR)

CSTF2 expression was evaluated by qRT-PCR, as previously described [16]. Total RNA was isolated from LIHC cells using the TRIzol reagent (#R0016). CSTF2 expression was measured using BeyoFast™ SYBR Green One-Step qRT-PCR Kit (#D7268S, Beyotime Biotechnology) referencing to the manufacturer’s instructions. β-Actin was used as the internal control. Relative CSTF2 expression was calculated using the 2^−ΔΔCt^ method [17]. The primer sequences were as follows: CSTF2 F: 5’-CCCAGTCTTTGGGTGGAGTT-3’, R: 5’-GGGTCTTGCATCGGCACTTG-3’; β-actin F: 5’-GATTCCTATGTGGGCGACGA-3’, R: 5’-AGGTCTCAAACATGATCTGGGT-3’.

### Western blot

Expression of CSTF2 and apoptosis-, metastasis- and pathway-related factors was detected using western blot, as previously described [18]. Total cellular protein extraction was carried out using RIPA lysis reagent (#P0013C) plus protease inhibitor cocktail (#P1005). The concentration of the extracted proteins was determined using the BCA protein assay kit (#P0012S). The proteins were then separated by SDS-PAGE and transferred onto PVDF membranes (#FFP24). After that, the membranes were blocked in 5% BSA at 25°C for 1 h, incubated with primary antibodies at 4°C for 16 h, and incubated with secondary antibody at 25°C for 2 h. Each step was followed by washing with TBST. The target bands were visualized using the BeyoECL Plus kit (#P0018S, Beyotime Biotechnology). Images were obtained using a bioimaging system (Bio-Rad) and analyzed using the ImageJ software.

Primary antibodies: CSTF2 (GTX114994, GeneTex), Bax (GTX109683, GeneTex), cleaved-caspase-3 (GTX86900, GeneTex), Bcl-2 (GTX100064, GeneTex), Claudin 1 (GTX134842, GeneTex), ZO-1 (GTX108613, GeneTex), MMP-9 (GTX100458, GeneTex), MMP-2 (GTX104577, GeneTex), N-cadherin (GTX112734, GeneTex), vimentin (GTX00942, GeneTex), p-AKT (ab38449, Abcam), AKT (ab8805, Abcam), p-PI3K (ab182651, Abcam), PI3K (ab288374, Abcam), p-mTOR (ab1093, Abcam), mTOR (ab137341, Abcam), GAPDH (GTX100118, GeneTex, CA, USA).

### Colony formation

Colony formation assay was performed as described previously [19]. After transfection with the indicated siRNAs or plasmids for 24 h, HCCLM3 and Huh7 cells (1.5 × 10^3^ cells/well) were plated in 6-well plates and maintained in complete DMEM for 14 days. After rinsing with PBS, the cells were fixed in methanol (#M188413) for 10 min and stained with 0.1% crystal violet (#C196471, Aladdin, China) for another 10 min. The colonies were captured and counted using a microscope.

### Cell proliferation

Cell proliferation was assessed using 5-ethynyl-2ʹ-deoxyuridine (EdU) proliferation assay (#R11053.9, RiboBio Biotechnology, China), as previously described [20]. After transfection, the Huh7 and HCCLM3cells (1 × 10^5^ cells/well) were maintained in DMEM containing EdU for 2 h. After washing, the cells were fixed in 4% paraformaldehyde (PFA, #P395744, Aladdin), destained in glycine (#G119904, Aladdin) and permeated in 0.5% TritonX-100 (#P0096, Beyotime Biotechnology). Then, 1 × Apollo® staining was performed in the dark at 25°C for 30 min, and the nuclei were stained with 1 × Hoechst33342 solution for another 30 min under the same conditions. Finally, the images were observed and captured under a microscope.

### Cell apoptosis

Cell apoptosis was evaluated utilizing an Annexin V-FITC Apoptosis Detection Kit (#C1062M, Beyotime Biotechnology), as previously described [21]. After transfection, HCCLM3 and Huh7 cells (5 × 10^4^ cells) were suspended in 195 μL annexin V-FITC binding solution. After that, 5 μL annexin V-FITC and 10 μL propidium iodide (PI) reagent were successively added and gently mixed. After incubation at 25°C in the dark for 15 min, the results were detected with a flow cytometer (BD Biosciences, USA).

### Cell migration and invasion

Cell migration and invasion were tested using transwell chambers (Corning), as previously described [22]. After transfection, HCCLM3 and Huh7 cells suspended in serum-free DMEM were added to the upper chambers, while complete DMEM was added to the lower chambers. The upper chambers used in the invasion assay were pre-coated with Matrigel (#354,234, Corning, USA). After culturing under 5% CO_2_ at 37°C conditions for 48 h, the migrated and invaded cells were fixed in PFA for 20 min and stained with 0.2% crystal violet for 10 min. Images were captured with a microscope and cell counting was performed using the IPP software.

### Pathway enrichment analysis

GSEA (version 4.0; http://software.broadinstitute.org/gsea/index.jsp) was performed to determine the potential pathway that participates in the regulation of CSTF2 in LIHC based on the TCGA-LIHC database.

### Statistical analysis

Data were obtained from three independent trials and are presented as mean ± standard deviation (SD). GraphPad Prism 8.0 was used for the statistical analyses. Differences among multiple groups and between two groups were analyzed using one-way ANOVA and Student’s *t*-test, respectively. Statistical significance was set at *P*< 0.05.

## Results

### CSTF2 was highly expressed in LIHC tissues and cells

**Figure 1.**
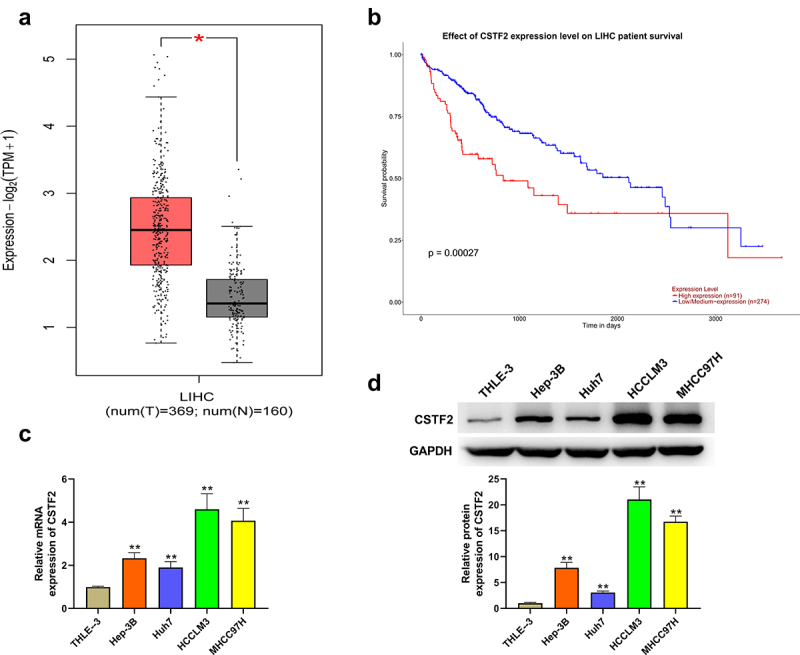
CSTF2 was highly expressed in LIHC tissues and cells.

First, the expression of CSTF2 in LIHC tissues and cells was assessed. Analysis of CSTF2 expression with data obtained from TCGA-LIHC database proved that CSTF2 levels in LIHC tissues were significantly higher than those in normal tissues (Figure 1a, *P* < 0.05). Besides, Figure 1b displays that patients with high CSTF2 expression had a lower overall survival probability (*P* < 0.001). Then, CSTF2 expression in LIHC cells was further determined to verify the results of bioinformatic analysis. Figures 1c and 1d showed that both the mRNA and protein levels of CSTF2 in LIHC cells were higher than those in THLE-3 cells (all *P* < 0.01). These data manifested that CSTF might function as an oncogene in LIHC.

(a) CSTF2 expression in LIHC and normal tissues was determined by GEPIA website with data obtained from TCGA-LIHC database. (b) Survival probability of LIHC patients was analyzed using Kaplan–Meier survival curve. (c) and (d) CSTF2 expression in LIHC cells were tested via western blot and qRT-PCR, respectively. *P < 0.05; **P < 0.01.

### CSTF2 overexpression exacerbated the malignant phenotypes in LIHC cells

**Figure 2.**
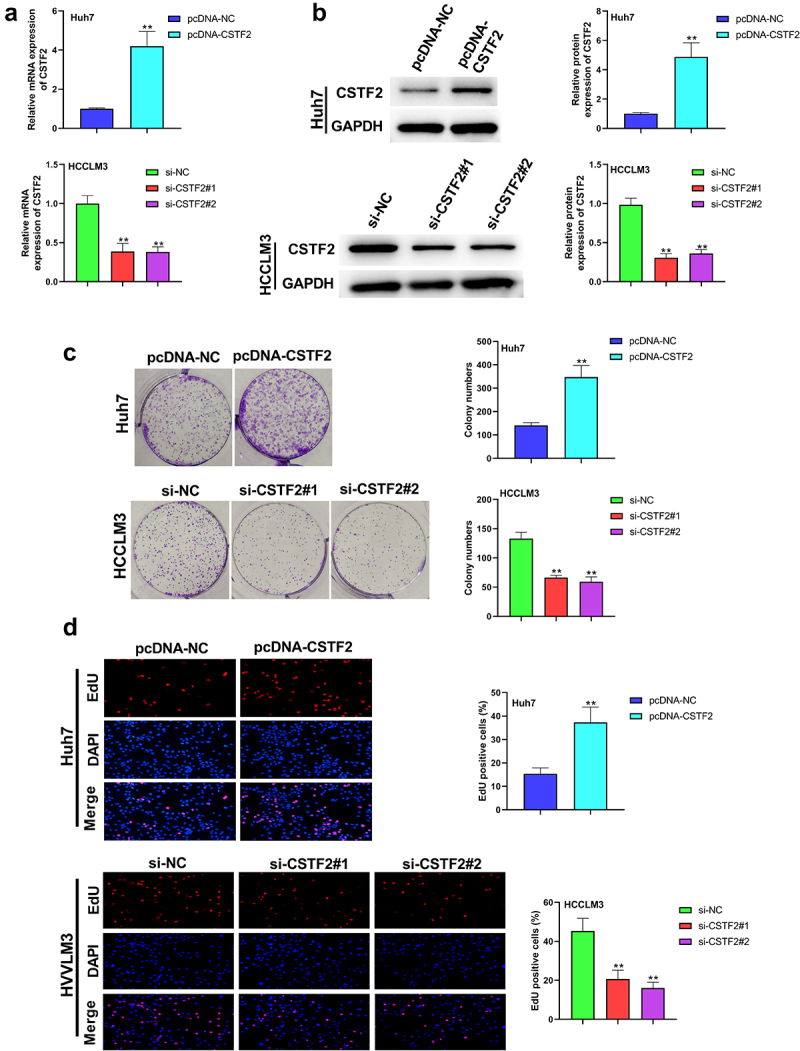
CSTF2 overexpression facilitated Huh7 cell colony formation and proliferation.

**Figure 3.**
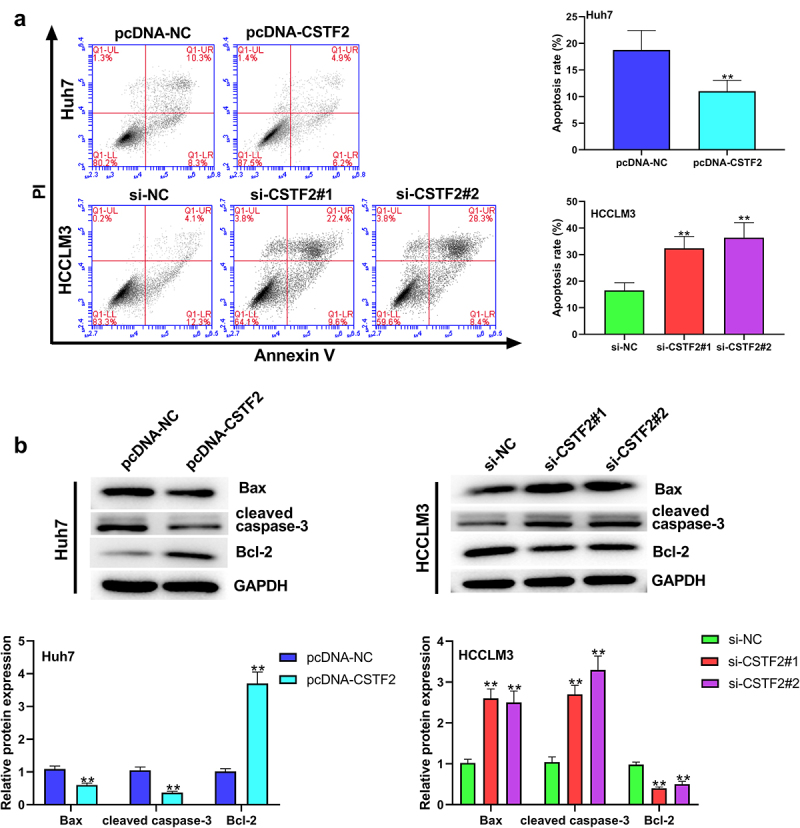
CSTF2 overexpression restrained Huh7 cell apoptosis.

**Figure 4.**
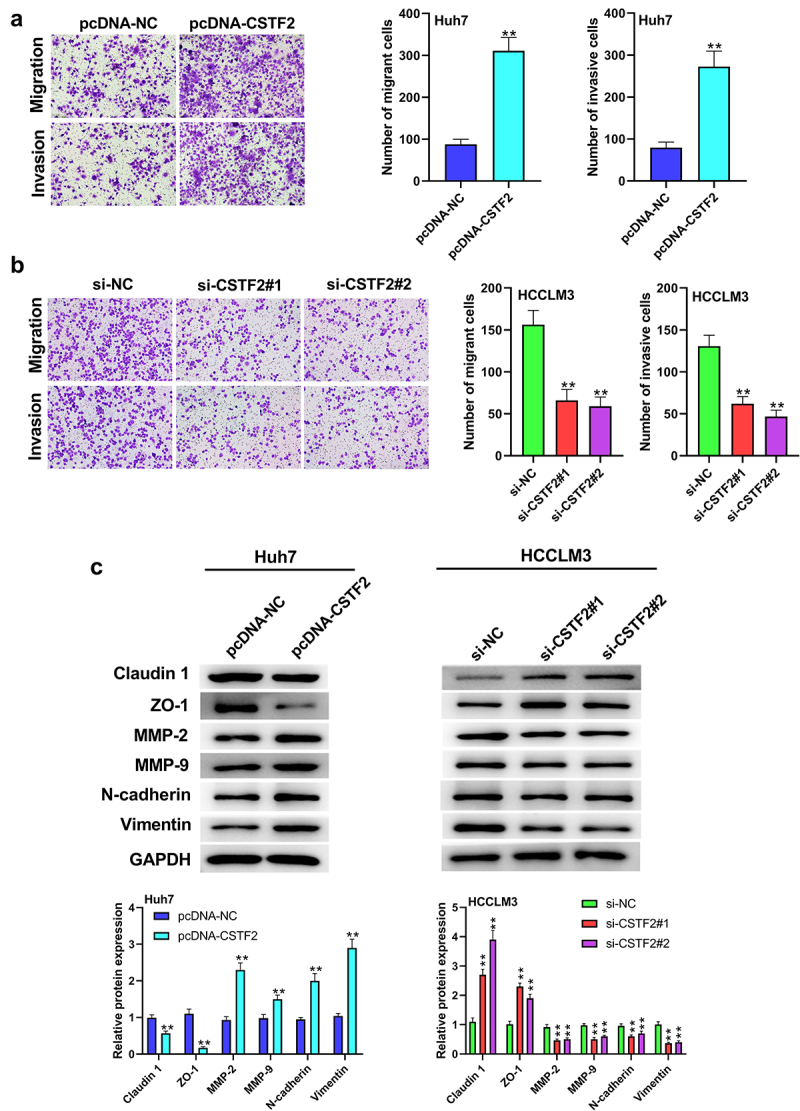
CSTF2 overexpression enhanced Huh7 cell migration and invasion.

Next, the effects of CSTF2 overexpression or knockdown on LIHC cells’ proliferation, apoptosis, migration and invasion were further investigated. Considering that CSTF2 expression was the lowest in Huh7 cells, and the highest in HCCLM3 cells, Huh7, and HCCLM3 cells were selected to carry out CSTF2 overexpression and knockdown assays, respectively. Figures 2a and 2b showed that transfection with pcDNA-CSTF2 notably upregulated CSTF2 expression in Huh7 cells both at mRNA and protein levels (both *P* < 0.01), while transfection with si-CSTF2#1 or si-CSTF2#2 both markedly suppressed CSTF2 expression in HCCLM3 cells (all *P* < 0.01). These results illustrated that CSTF2 overexpression and knockdown were successfully achieved in Huh7 and HCCLM3 cells, respectively. Thereafter, the influences of the abnormal CSTF2 expression on the phenotypes of the transfected Huh7 and HCCLM3 cells were determined. Figures 2c and 2d present that CSTF2 overexpression observably facilitated colony formation and proliferation of Huh7 cells (both *P* < 0.01). Figure 3a shows that Huh7 cell apoptosis was distinctly repressed by CSTF2 overexpression (*P* < 0.01), which was validated by the reduced expression of apoptosis-related cleaved-caspase-3 and Bax, and the elevated expression of Bcl-2 (all *P* < 0.01). Figure 4a presents that the invasion and migration of Huh7 cells were both remarkably augmented by CSTF2 overexpression (both *P* < 0.01), which was confirmed by the decreased expression of Claudin 1 and ZO-1, and increased expression of MMP-9, MMP-2, vimentin, and N-cadherin (Figure 4c, all *P* < 0.01). However, CSTF2 knockdown had the opposite effect on HCCLM3 cells (Figures 2c-4c, all *P* < 0.01). These findings illustrated that high expression of CSTF2 promoted the development of malignant phenotypes in LIHC cells.

A and B. pcDNA-CSTF2 or pcDNA-NC was transfected into Huh7 cells to overexpress CSTF2. si-CSTF2#1, si-CSTF2#2 and si-NC were respectively transfected into HCCLM3 cells to achieve CSTF2 knockdown. C. Colony formation capacity of these transfected cells were assessed via colony formation assay. D. Cell proliferation of the transfected cells were evaluated via EdU assay. **P < 0.01.

A. Cell apoptosis of the transfected Huh7 and HCCLM3 cells were measured using a Annexin V-FITC Apoptosis Detection Kit. B. Expression of apoptosis-associated Bax, Bcl-2 and cleaved-caspase-3 in the transfected cells was tested via western blot. **P < 0.01.

A and B. Cell invasion and migration of the transfected Huh7 and HCCLM3 cells were tested via transwell assay. C. Expression of metastasis-associated Claudin 1, ZO-1, MMP-2, MMP-9, Vimentin and N-cadherin in the transfected cells was tested via western blot. **P < 0.01.

### CSTF2 overexpression notably activated PI3K/AKT/mTOR pathway

**Figure 5.**
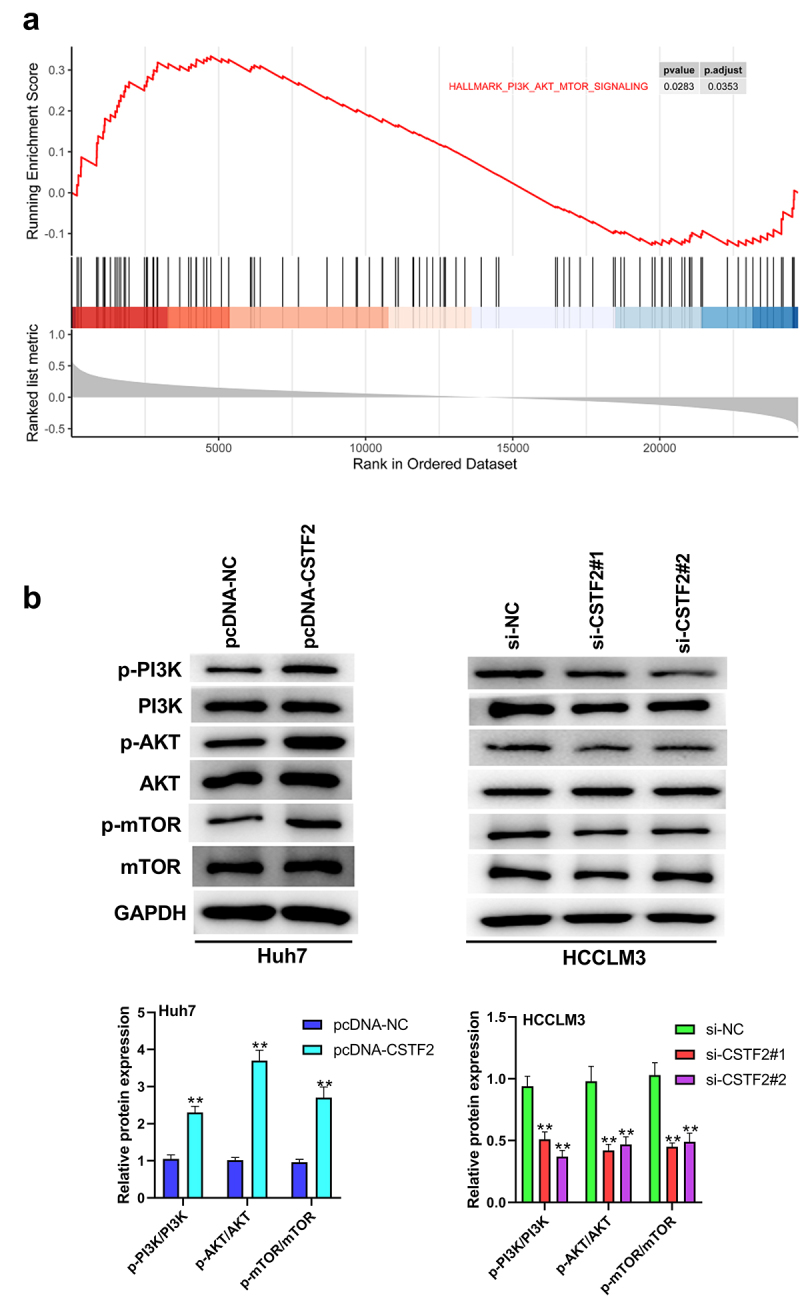
PI3K/AKT/mTOR pathway participated in the regulation of CSTF2 in LIHC.

Pathway enrichment analysis revealed that the PI3K/AKT/mTOR pathway was closely associated with CSTF2 expression, indicating that CSTF2 might regulate LIHC progression via the PI3K/AKT/mTOR pathway (Figure 5a). To test this hypothesis, the expression of PI3K/AKT/mTOR pathway-associated factors in transfected Huh7 and HCCLM3 cells was analyzed. Figure 5b presents that the ratios of p-AKT/AKT, p-PI3K/PI3K and p-mTOR/mTOR were markedly augmented by CSTF2 overexpression (all *P* < 0.01), indicating that the PI3K/AKT/mTOR pathway was activated by CSTF2 overexpression. However, CSTF2 knockdown lowered these ratios, indicating that this pathway was deactivated (all *P* < 0.01). These results indicated that CSTF2 overexpression might exacerbate the malignant phenotypes of LIHC cells by activating the PI3K/AKT/mTOR pathway.

A. Pathway enrichment analysis was carried out using GSEA analysis based on TCGA-LIHC database. B. Expression of PI3K/AKT/mTOR-associated mTOR, p-mTOR, AKT, p-AKT, PI3K and p-PI3K in the transfected cells was tested via western blot. **P < 0.01.

### The effects of CSTF2 on the development of malignant phenotypes in LIHC cells were reversed by 740Y-P or Wortmannin

**Figure 6.**
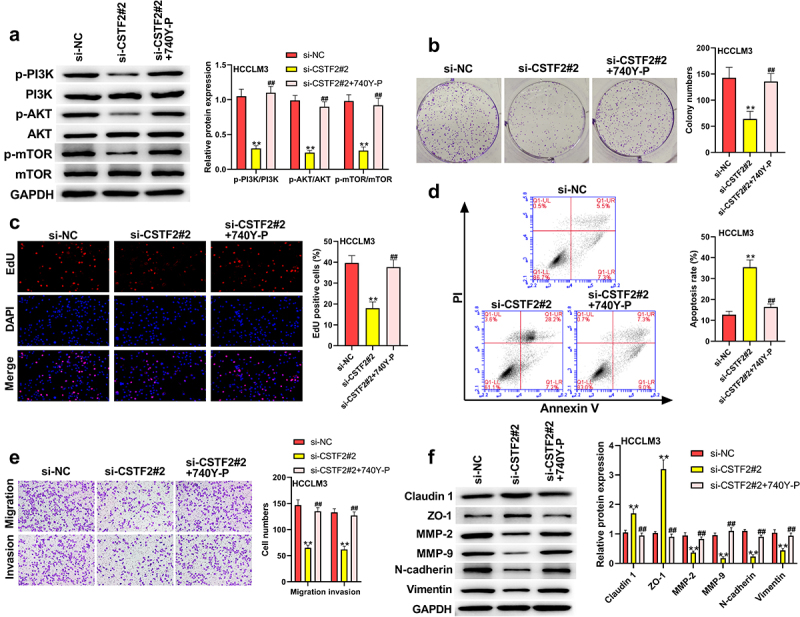
740Y-P, a PI3K activator, reversed CSTF2 knockdown-triggered tumor suppressing effects on HCCLM3 cells.

**Figure 7.**
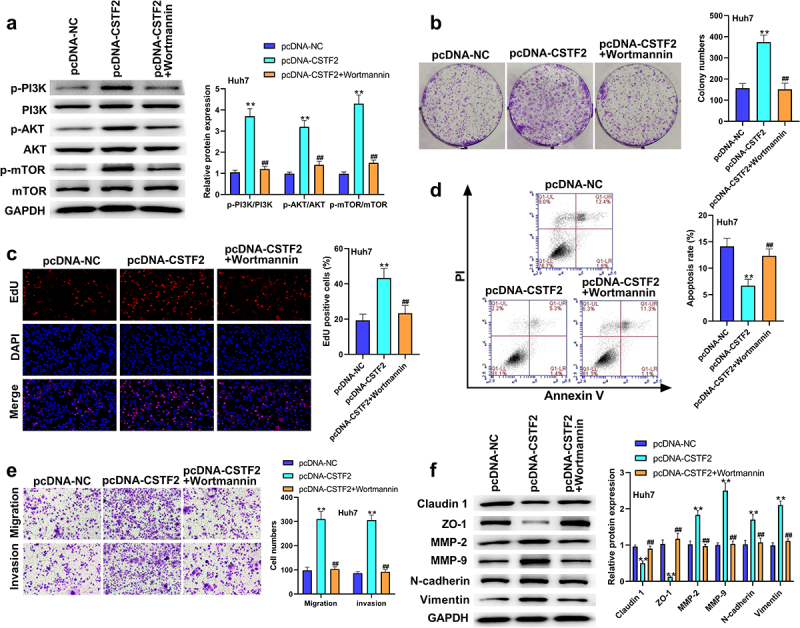
Wortamnnin, a PI3K inhibitor, reversed CSTF2 overexpression-caused tumor promoting effects on Huh7 cells.

To further validate whether the PI3K/AKT/mTOR pathway participates in the regulation of CSTF2 in LIHC, transfected HCCLM3 cells or Huh7 cells were treated with the PI3K activator 740Y-P or PI3K inhibitor Wortmannin, respectively. Figure 6a shows that 740Y-P treatment significantly removed CSTF2 knockdown-triggered deactivation of the PI3K/AKT/mTOR pathway (all *P* < 0.01). Figure 6b and 6c presented that CSTF2 knockdown-induced inhibition of HCCLM3 cell colony formation and proliferation was distinctly reversed by 740Y-P treatment (both *P* < 0.01). Figure 6d displays that the elevated apoptosis rate induced by CSTF2 knockdown was notably abolished by 740Y-P treatment (*P* < 0.01). Moreover, CSTF2 knockdown-triggered suppression of HCCLM3 cell invasion and migration was reversed by 740Y-P treatment, which was validated by variations in metastasis-associated proteins (Figure 6e and 6f, all *P* < 0.01). Similarly, Wortmannin treatment significantly abolished CSTF2 overexpression induced activating effects on PI3K/AKT/mTOR pathway (Figure 7a, all *P* < 0.01). In addition, CSTF2 overexpression induced promoting effects on colony formation, proliferation, migration, and invasion, or inhibiting effect on apoptosis of Huh7 cells were abolished by Wortannin treatment (Figure 7b–7f, all *P* < 0.01). Collectively, these data demonstrated that CSTF2 could promote the development of malignant phenotypes in LIHC cells via activation of the PI3K/AKT/mTOR pathway.

A. The transfected HCCLM3 cells were treated with 740Y-P reagent. Then, expression of PI3K/AKT/mTOR-associated mTOR, p-mTOR, AKT, p-AKT, PI3K and p-PI3K in the transfected cells was tested via western blot. B. Colony formation capacity of these transfected cells were assessed via colony formation assay. C. Cell proliferation of the transfected cells were evaluated via EdU assay. D. Cell apoptosis of the transfected HCCLM3 cells were tested via a Annexin V-FITC Apoptosis Detection Kit. E. Cell invasion and migration of the transfected HCCLM3 cells were assessed by transwell assay. C. The protein expression of metastasis-associated Claudin 1, ZO-1, MMP-2, MMP-9, Vimentin and N-cadherin in the transfected cells was tested via western blot. **P < 0.01, ^##^*P* < 0.01.

A. The transfected Huh7 cells were treated with Wortamnnin. Then, expression of PI3K/AKT/mTOR-associated mTOR, p-mTOR, AKT, p-AKT, PI3K and p-PI3K in the transfected cells was determined by western blot. B. Colony formation capacity of the transfected Huh7 cells were assessed by colony formation assay. C. EdU assay was used to detect cell proliferation of the transfected Huh7 cells. D. Cell apoptosis of the transfected Huh7 cells were tested by using a Annexin V-FITC Apoptosis Detection Kit. E. Cell invasion and migration of the transfected Huh7 cells were assessed using transwell assay. C. The protein expression of metastasis-associated Claudin 1, ZO-1, MMP-2, MMP-9, Vimentin and N-cadherin in the transfected Huh7 cells was tested using western blot. **P < 0.01, ^##^*P* < 0.01.

## Discussion

LIHC is the most common form of primary liver cancer, with mortality ranking third among malignant tumors in 2020 [1]. In this study, we discovered that CSTF2 was highly expressed in LIHC. Besides, CSTF2 overexpression induced malignant phenotypes, such as inhibited apoptosis and facilitated proliferation, migration, and invasion of Huh7 cells. Conversely, CSTF2 knockdown impeded the development of these malignant phenotypes in HCCLM3 cells. The PI3K/AKT/mTOR pathway has been shown to be involved in the regulation of CSTF2 in LIHC, and was proven to be deactivated by CSTF2 knockdown or be activated by CSTF2 overexpression. Further experiments validated that 740Y-P treatment reversed CSTF2 knockdown-triggered tumor-repressing effects on HCCLM3 cells. Similarly, Wortmannin treatment also abolished CSTF2 overexpression-induced tumor promoting effects on Huh7 cells. These findings indicated that CSTF2 might serve as an oncogene in LIHC via activating the PI3K/AKT/mTOR pathway.

CSTF2, a component of CSTF, was found to function as an oncogene in multiple tumors. For instance, CSTF2 is upregulated in colon adenocarcinoma, liver cancer, uterine corpus endometrioid carcinoma, bladder urothelial carcinoma, breast invasive carcinoma, and lung adenocarcinoma [23–26]. Cells depleted of CSTF2 experienced apoptotic death and cell cycle arrest [13,27,28]. Moreover, CSTF2 activation has been shown to promote oncogenic activities in urothelial carcinoma of the bladder [14]. Furthermore, CSTF2 was found to be highly expressed in NSCLC cells, and was deemed to regulate NSCLC progression by modulating the 3’-UTR length of cancer-associated genes [29]. A previous study demonstrated that the shortening 3’-UTR isoform could escape miRNA-targeted inhibition and exert crucial carcinogenic roles in pathogenesis [14,30]. In this study, we discovered that CSTF2 was highly expressed in LIHC cells, and its expression was associated with poor prognosis in LIHC patients. Furthermore, CSTF2 overexpression facilitated LIHC cell colony formation, proliferation, invasion, and migration, while repressing cell apoptosis. CSTF2 knockdown showed the opposite effects. These results were in accordance with the aforementioned studies, which indicated that CSTF2 serves as an oncogene in LIHC.

The PI3K/AKT/mTOR pathway has been demonstrated to participate in various cellular processes, such as cell survival, proliferation, differentiation, metastasis, apoptosis, metabolism, and angiogenesis [31,32]. Members of this pathway are often activated or mutated in cancer [33]. A previous study showed that activation of the PI3K/AKT pathway augmented squamous cell carcinoma cell invasion and metastasis [34]. Another study revealed that the anticancer effects of anemoside B4 were achieved in LIHC through deactivation of the PI3K/AKT/mTOR pathway [35]. Besides, Song *et al*. revealed that lncRNA RHPN1-AS1 enhanced LIHC cell proliferation, invasion, and migration via activation of the PI3K/AKT/mTOR pathway [36]. Sun and his colleagues found that DUXAP10 knockdown impeded colony formation capacity and proliferation, while facilitating apoptosis of LIHC cells by inhibiting the PI3K/AKT pathway [37]. This evidence demonstrates that there is a correlation between the development of malignant phenotypes in tumor cells and activation of the PI3K/AKT/mTOR pathway. Moreover, GSEA analysis confirmed that PI3K/AKT/mTOR pathway was closely associated with CSTF2 expression, suggesting that CSTF2 might regulate LIHC progression via PI3K/AKT/mTOR pathway. Our results confirmed that the PI3K/AKT/mTOR pathway was notably activated by CSTF2 overexpression, whereas it was inhibited by CSTF2 knockdown. Thus, these results are in line with those of previous investigations. To elucidate potential regulatory mechanisms, the rescue experiments were performed. Following experiments revealed that CSTF2 knockdown-induced tumor repressing effects or CSTF2 overexpression-induced tumor promoting effects were reversed by 740Y-P treatment or Wortamnnin, respectively. These results illustrated that CSTF2 could promote the proliferation, migration and invasion, as well as inhibit the apoptosis in LIHC cells through activating the PI3K/AKT/mTOR pathway.

## Conclusions

This study demonstrated that CSTF2 might function as an oncogene in LIHC and that CSTF2 could exacerbate the malignant phenotypes of LIHC cells by activation of the PI3K/AKT/mTOR pathway.

## Data Availability

The datasets used and analyzed during the current study are available from the corresponding author.
